# Impact of Mechanical Removal on the Regeneration and Colonization Abilities of the Alien Aquatic Macrophyte *Egeria densa*

**DOI:** 10.3390/life13102004

**Published:** 2023-10-02

**Authors:** Gabrielle Thiébaut

**Affiliations:** Université de Rennes, UMR 6553 CNRS ECOBIO, 263 Avenue du Général Leclerc, 35042 Rennes, France; gabrielle.thiebaut@univ-rennes.fr

**Keywords:** aquatic plant, mass development, management, biological traits, mesocosms

## Abstract

The development of aquatic plant beds can obstruct boat traffic, hinder the practice of water activities, and impact the functioning of freshwaters. In order to mitigate their effects, mechanical removal is often the preferred management solution. The objective of this study was to test, in mesocosms, the effect of frequency (none, one, and two cuts) and cutting dates (May and/or July) on the regeneration and colonization capabilities of the aquatic plant *Egeria densa*, an invasive alien species in France. The cutting date had no effect on the capabilities of *E. densa*, but the two cuts significantly reduced the plant’s biomass. Removal produced numerous fragments, which exhibited very high survival and anchoring rates. However, summer removal produced fragments with lower regeneration and colonization abilities compared to fragments from spring cutting. Mechanical removal only temporarily reduced the biomass of the aquatic plant beds and could promote the formation of new beds from the fragments generated by management and dispersed by water flow.

## 1. Introduction

Some Invasive Aquatic Plant species (IAP) are ecosystem engineers that fundamentally and irreversibly change the physical and biotic habitat of freshwaters [1]. They can limit light therefore oxygen depletion, alter biological communities, resulting in food web changes. They are expected to increase in frequency due to global changes, and as such, the problems they create will become worse. Their development induces economic costs because they can deleteriously affect recreational activities (boating, fishing, swimming, and other water sports), interfering with hydroelectric generation and drainage, and increasing the risk of flood in the valley [2,3].

Among the invaders, the Brazilian waterweed *Egeria densa* Planchon (Hydrocharitaceae) is a submersed, freshwater perennial macrophyte native to subtropical regions of South America. Being one of the most common plants for aquaria [4] and often used for biochemical and physiological investigations, *E. densa* has been widely distributed around the world. In France, *E. densa* has been in cultivation since at least 1919 [5], was released into the wild in 1960, and has then spread along the entire Atlantic coast [6]. *E. densa* has the tendency to dominate its environment by vigorous growth and it often produces dense monospecific mats that can obstruct channels, marinas, and irrigation systems and disrupt navigation. Among the problems it creates are restriction water movement, reservoir flow interruption, water quality alteration, and biodiversity impact [7]. *E. densa* has been reported to outcompete native aquatic plants by blocking light needed by other plants and to adversely affect fish communities [8,9]. Dense vertical stands produce anoxic conditions and trap sediments in the system, while dieback can increase nutrient loads to the water [8]. They also increase thermal stratification [7]. The increases in water clarity and temperature can promote the further growth and spread of *E. densa* itself, while facilitating invasion by other alien species, particularly fishes [10]. 

The Brazilian waterweed *E. densa* is difficult to detect at early stages of invasion, and therefore control or eradication actions often start when the plant is already well-established. The use of herbicides and biological control agents (except widely stocked grass carp) for the control of IAPs are not permitted (except in the UK) in European countries [11]. Only a few studies focused on the regeneration and colonization abilities of IAPs after cutting [12,13]. The successful management of *E. densa* requires comprehensive knowledge of the biology of the invader and its ability to regrow after stress such as removal or after a disturbance like flooding. To evaluate the impact of the period and the frequency of cutting on *E. densa* biomass and precisely investigate the process of regrowth and the ability of propagule release to regenerate and colonize new sites, an approach based on measurements of morphological traits in experimental outdoor mesocosms was used. The first hypothesis was that the summer cut would stimulate plant growth and a cutting in summer had a lower effect than a spring cut. The premise of this hypothesis is that shoots with roots develop to the surface of the water when the bottom-water temperature increases above 15 °C [14]. The second hypothesis was that the rooting abilities of the fragments would be higher in summer than in spring as the root crown of *E. densa* developed simultaneously as the plant biomass increased [14]. A biomass peak was reached in August [15]. The main goals of this study were to determine: (i) the impact of the number and period of cutting on plant biomass and the most appropriate period to proceed so as to reduce the regrowth of *E. densa* in terms of biomass, (ii) and the ability of the *E. densa* fragments to regenerate and colonize new sites after the removal. 

## 2. Material and Methods

### 2.1. Plant Collection

The biological model, *E. densa*, was collected from a pond near Rennes (N: 48°01′15″; W: 01°43′81″) in Brittany, northwestern France. One hundred ramets, each consisting of a stem with a single apex, were collected in mid-April, from the same individual. Back at the laboratory facility, we selected fragments with an apex, excluding lateral branches and roots. All fragments were 10 cm in length. The size, fresh weight, and number of buds on each fragment were measured. The fragments were kept indoors in tap water for three days before being transferred to outdoor mesocosms.

### 2.2. Experimental Design

Five mesocosms (1.50 m length × 1.20 m width × 0.50 m depth) located at the experimental garden (University of Rennes 1, France) were used. Each mesocosm was divided into six sub-units using frost protection fabric to allow water circulation Water was actively circulated with a pumping system set up to maintain a water depth of 0.50 m in each mesocosm. Circulating water was drawn to and from a tank adjacent to the mesocosms.

From April to mid-September, the water quality and water depth in each mesocosm were monitored monthly (Table S1). Water temperature and conductivity were measured once a week in the morning in each mesocosm (YSI Professional Plus, Xylem Inc., Yellow Springs, OH, USA). Water samples were collected from the tanks about once a month from April to September to determine pH using a pH probe. Nutrient concentrations were assessed for: NO_3_, PO_4_^3−^ (colorimeter tests with reagents HI-93728, HI-93713, photometer HI-83200, Hanna Instrument, Woonsocket, RI, USA), and NH_4_^+^ (spectrophotometry). Liquid fertilizer (NPK 4-6-6) was added to the water tanks three times during the experiment to maintain non-limiting nutrient conditions (see Supplementary Table S1 for data). Two experiments were performed.

**Experiment** **1.** Effect of the Frequency of Cutting on the Biomass of E. densa

We defined six modalities: (1) control, no cutting throughout the experiment; (2) one cutting in spring (May); (3) one cutting in summer (July); (4) one cutting in May and one cutting in July; (5) none cutting in May (called control May); (6) none cutting in July (called control July). Within each mesocosm, each sub-unit was randomly assigned to one of the six modalities, ensuring each mesocosm contained all modalities. Within each sub-unit, we placed 3 pots filled with 2 cm of sand over 1 cm of potting soil (NPK 16-7-15) and planted 3 fragments of *E. densa* in each pot. The plants were acclimated to these conditions for four weeks at ambient temperature from April to May. The vegetation was then cut just above the substratum with scissors and removed from the sub-unit. Two parameters were tested:

- The number of cuts: zero, one, or two cuts.
- The removal date: one removal was conducted in May (after one month), one in July (after three months), and one removal was carried out in May and in July (two treatments), respectively.

To investigate the effect of the no-management option on plant growth, we measured several morphological traits in the control sub-units. In May, July, and at the end of the experiment, we collected plants. We measured growth-related traits such as stem length and fresh above-ground mass. We also counted the number of roots, the mean size of roots, the number of buds, and lateral branches. Afterward, we weighed the shoots (fresh mass), dried them (for 1 week at 70 °C), and then reweighed them (dry mass). We calculated the “RGR”, the Relative Growth Rate [16]:

$$RGR=\frac{\ln\left(m_{2}\right)-ln(m_{1})}{t_{2}-t_{1}}$$

where m_1_ and m_2_ refer to plant mass at time 1 (at the beginning of the experiment in April) and time 2 (at the end of the experiment in May, July, or September) (*t*_1_ and *t*_2_, respectively).

To compare the effect of the frequency and date of cutting, the total dry biomass was measured at the end of the experiment.

**Experiment** **2.** Recolonization and Regeneration Abilities of Floating Fragments

After the first cut in May, we collected five fragments with an apex in each sub-unit. These five free-floating fragments of *E. densa* were introduced into an empty sub-unit. Each sub-unit was filled with 2 cm of sand above 1 cm of potting soil (NPK 16-7-15) as substrate. The experiment (n = 5) lasted for ten weeks, from mid-May until mid-July.

Two parameters were recorded: (1) survival rate, measured as the percentage of surviving fragments (rooted and floating in water); (2) anchorage rate (=rooting efficiency), measured as the percentage of fragments that anchored themselves into the sediment. The anchorage rate of the fragments was expressed as FNR/FN0 × 100%, where FNR is the number of fragments rooted into the sediment, and FN0 is the number of original fragments [12]. When a new bud developed and detached from the fragment, it was counted as a new individual. The fragments were checked weekly for anchorage and survival rates, and dead plant material was removed from the tanks. Plants were removed at the end of the experiment (mid-July), and growth was evaluated using a trait approach. For each introduced fragment, we measured stem length, and the number of lateral branches, roots, and buds after ten weeks. To test whether the fragments allocated their energy to apical growth and/or their vigor, we measured their biomass. The fresh biomass was weighed for each plant individual.

A similar protocol was applied after the removal in July.

### 2.3. Statistical Analyses

All analyses were performed using R 4.0.0 [17]. To test the seasonal growth of plants in the control sub-units and the effect of cutting on the biomass of *E. densa*, a one-way ANOVA was conducted. The traits ‘number of lateral branches, number of buds, and number of roots were log-transformed to assess data homoscedasticity and normality of residuals using Levene’s tests. ANOVA was followed by post-hoc Tukey’s tests. All analyses were conducted at a *p* < 0.05 level of significance. 

Two analyses were conducted to test the differences in traits based on the season (spring-summer), the timing of the experiment (beginning-end), and their interaction. An ANOVA followed by Tukey’s test (pairwise comparison) was performed if the data was normally distributed. The Levene statistic was used to test for the equality of group variances. Otherwise, a Generalized Linear Model (GLM) assuming Poisson-distributed residuals was utilized (the model was transformed using the logarithm base 10). The Wald test was used to compare the coefficients of the linear model pairwise. For the anchoring rate, where the data were distributed into two values (0 or 1), a GLM was also used, assuming the residuals followed a binomial distribution. When the data were not normally distributed (Shapiro–Wilk *p* < 0.05), differences were assessed with Kruskal–Wallis as a post hoc comparison.

## 3. Results

### 3.1. Effect of No Management on the Plant’s Growth

**Figure 1:**
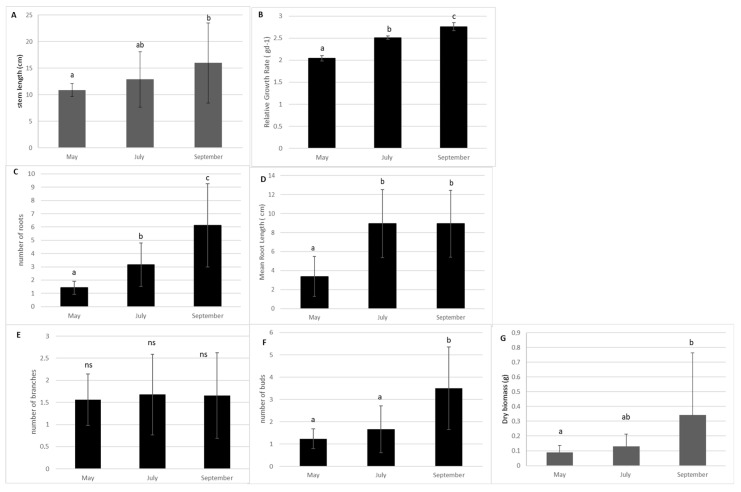
Measurement of morphological traits of the shoots of *E. densa* according to month in the control sub-units (Mean value and standard deviation). (**A**) stem length; (**B**) Relative Growth Rate (RGR); (**C**) number of roots; (**D**) mean length root; (**E**) number of lateral branches and (**F**) number of buds. (**G**) Relative Growth Rate. Different small letters indicate significant differences in the traits at level *p* < 0.05; ns: no significance.

In the absence of management, the stem length and the Relative Growth rate of *E. densa* in the control sub-units significantly increased from spring to the end of September (*p*-value = 0.033 and *p*-value = 0.003 respectively, KW). Additionally, the dry biomass and bud production showed a strong increase after July (*p* = 0.0014; *p* > 0.0001, respectively). The production of lateral branches remained stable (Figure 1). Root production increased from May to September (*p* > 0.0001). The mean size of the roots increased from May to July and remained stable thereafter (*p* = 0.0013, Figure 1).

### 3.2. Impact of the Number and Period of Cutting on Dry Biomass 

**Figure 2:**
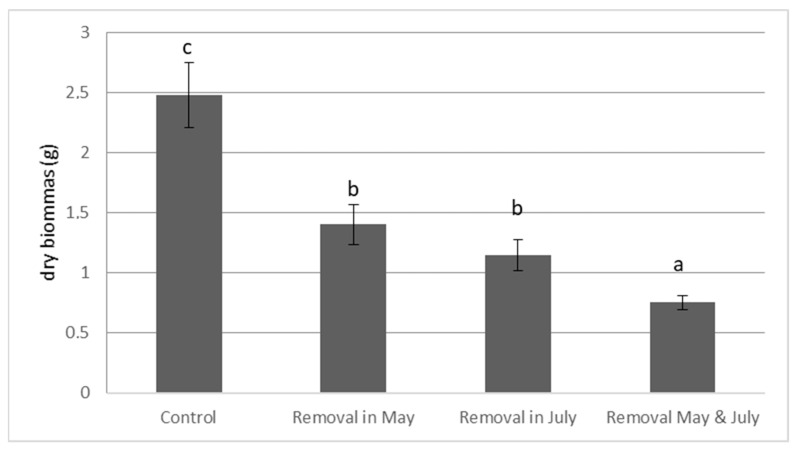
Effect of the frequency and of the date of cutting on the final dry biomass of *E. densa*. (mean value and standard deviation). Different letters indicate significant differences in the traits at level *p* < 0.05.

The removal date had no significant effect on the final dry biomass (DW) of *E. densa* (Figure 2). However, the frequency of cutting had a significant effect (*p*-value = 0.004). Two removals significantly reduced the final dry plant biomass (Control: DW = 2.480 g ± 0.273; Two cuts: DW = 0.753 g ± 0.058, Figure 2). After two cuts, the final dry biomass (DW) of *E. densa* was significantly lower than after one cut (Figure 2).

### 3.3. Effect of the Removal Date on Fragment’s Regeneration and Colonization Success

**Figure 3:**
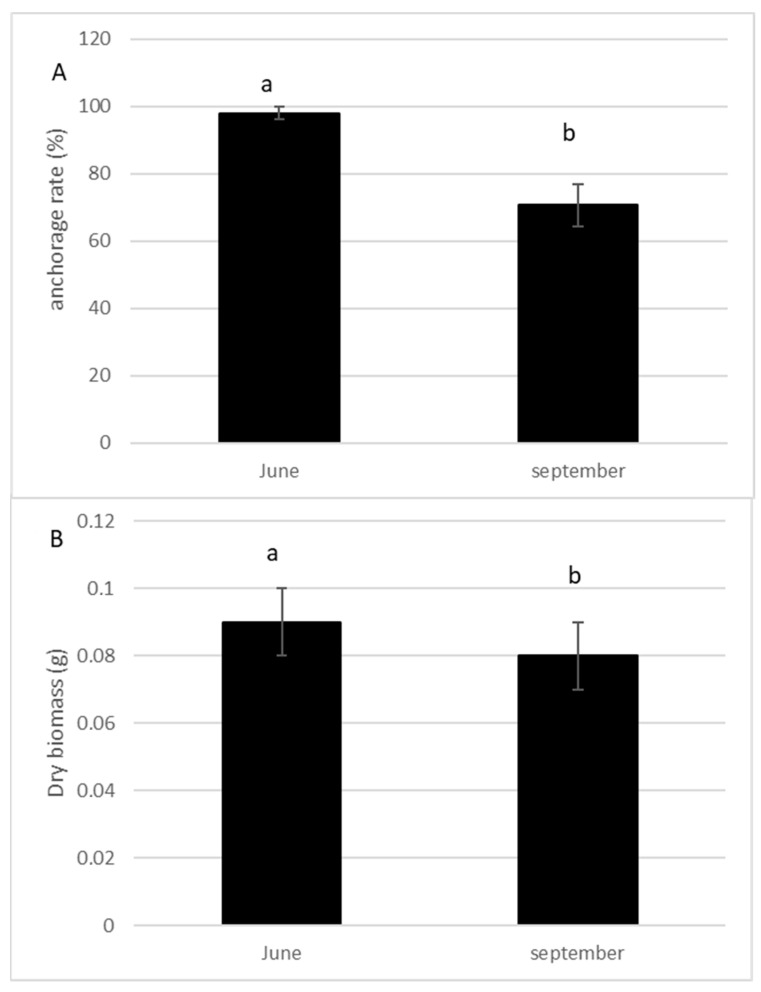
Percentage of anchorage (**A**) and dry biomass (**B**) of the fragments of *E. densa* one month after the cut either in May or in July (mean value and standard deviation). Different letters indicate significant differences in the traits at level *p* < 0.05.

**Figure 4:**
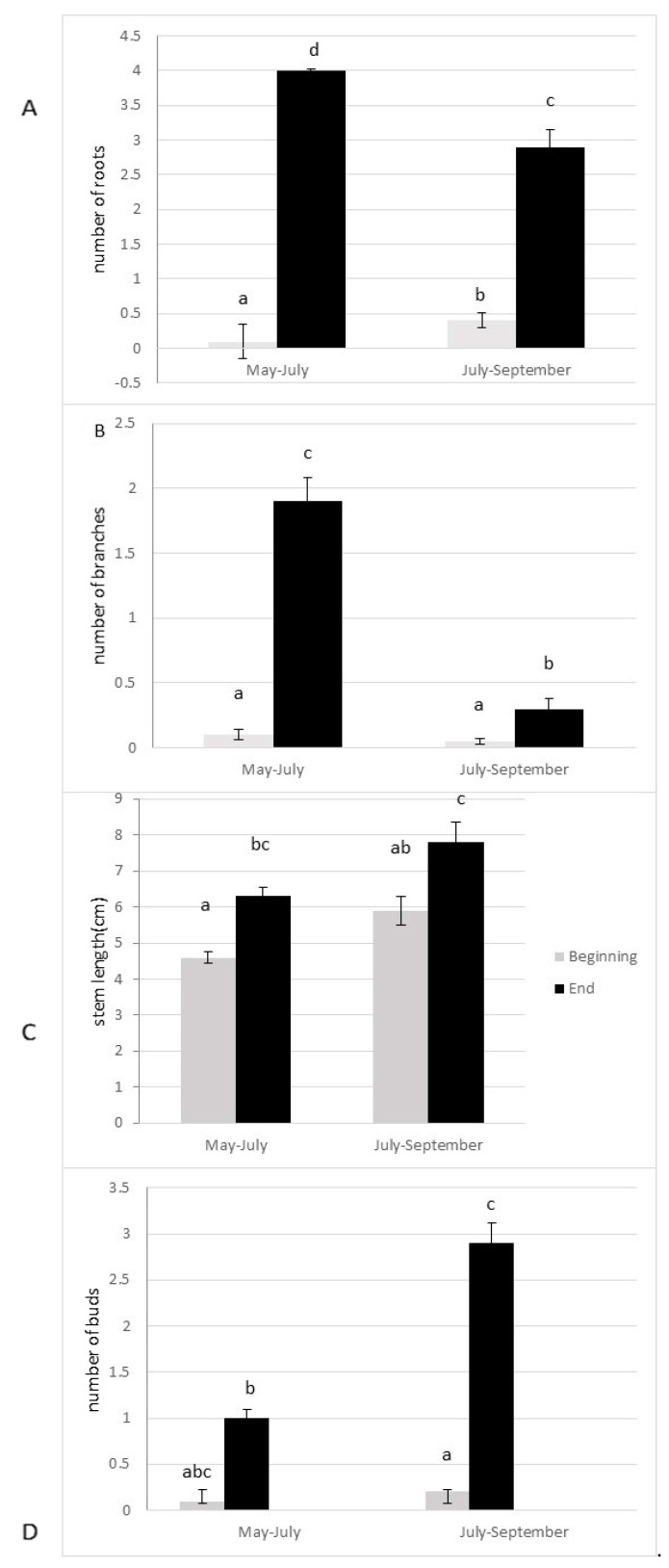
Colonization ability ((**A**) number of roots) and regeneration ability ((**B**) number of lateral branches; (**C**) stem length; (**D**) number of buds) of the fragments of *E. densa* after ten weeks (mean value and standard deviation). Different letters indicate significant differences in the traits at level *p* < 0.05.

The anchorage rate of fragments and dry biomass was significantly higher in spring than in summer (*p* = 0.004; *p* = 0.005, respectively, Figure 3A,B). The number of lateral branches, roots, and buds were higher (*p* > 0.0001, Figure 4), and the fragment size also significantly increased (*p* > 0.0001, Figure 4) 10 weeks after the cut, regardless of the date of the cut. However, a strong seasonal effect was observed for the tested traits (Table 1). Fragments produced by cutting in May exhibited better colonization ability (number of roots) and regeneration potential (higher anchorage rate, higher dry biomass, higher production of lateral branches) than fragments produced in July (Figure 3 and Figure 4).

## 4. Discussion

### 4.1. Impact of Frequency and Date of Removal on E. densa Biomass 

This study in outdoor mesocosms demonstrated that cutting reduced the final dry mass of *E. densa*. Despite the cutting, the plant continued to grow, but with reduced vigor compared to the control plants. These results suggest that cutting represents stress for the plant, resulting in a partial reduction in vigor and uncompensated mass loss. More unexpectedly, the final dry biomass did not significantly differ after summer or spring removals. The first hypothesis that the summer cut stimulated plant growth, was not validated. However, control plants exhibited a higher growth rate RGR in summer than in spring. After cutting, plants were unable to stimulate their apical growth. A difference in rooting activity could be attributed to the absence of the apical meristem. The growth of *E. densa* was lower for shoots without an apex compared to those with an apex [18]. Without an apex, plants invested in lateral growth and branch production (vigor) rather than apical growth. This strategy could explain the similar biomass after one cut in May and July. There was no appropriate period to proceed to the reduction of the regrowth of *E. densa* in terms of biomass. 

The results of this study confirmed the limited effect of one removal on the plant biomass. Cutting in either spring or summer reduced the final biomass of the plant, and the impact of management on the plant was reinforced when two cuts were performed during the year. One cut reduced the biomass by 44%, and the biomass of *E. densa* decreased by 83% after two cuts. Two cuts could eliminate the double nodes; these very short internodes form a meristematic region from which new buds can develop. These results are consistent with few studies found in the literature with *Elodea* sp. as biological models [19,20]. A previous study [19] showed in laboratory conditions that one cut did not significantly reduce the plant’s length of *Elodea canadensis* Michx., but decreased its biomass by 41%, while two cuts decreased its length by 44% and its biomass by 59%. Similarly, a field study [20] demonstrated that two removals significantly reduced the biomass of *Elodea nuttallii* Saint John. Therefore, cutting at two dates appears to be an effective solution for IAP management. However, despite the efforts made, removal led only to a partial eradication of *E. densa*. Although two cuttings showed better results than one cut, two mechanical removals per year are not economically possible, given the cost of this management. However, these results in outdoor mesocosms must be taken with caution as they were obtained in experimentally controlled conditions. 

A field survey of *E. densa* biomass after mechanical removal did not show a reduction in biomass in the river Vendée [21]. This difference in results between the field and the mesocosm experiment could be explained by unequal cutting pressure in the field, whereas all shoots underwent the same cut in mesocosms; the boat cannot reach all plants. The boat can reach plants located at a maximum depth of 1.20 m, whereas *E. densa* has been found as deep as 3.20 m [22]. Moreover, it cannot operate when the depth is less than one meter [22], which limits the effectiveness of cutting. 

The effects of removal are of short duration on the reduction of the biomass of *E. densa*. More research is required to understand the long-term impacts of repeated removals on the plant’s fitness and the importance of this management on the dispersal and colonization capacity.

### 4.2. Regeneration and Colonization Abilities of Fragments 

The survival rate of propagules, as well as their regeneration and colonization abilities, were high. The survival rate was 100%, a rate similar was found previously [18]. When a shoot sinks to the bottom after the removal, a new root crown may develop at one or several double nodes along the new shoot. The shoot rooted generally very quickly and found a high rate of rooting (=88%). The ability to regenerate from small stem fragments means that repeated removal could promote a secondary invasion. The colonization abilities (anchoring rate, number of roots) and regeneration abilities (number of branches, dry weight) of fragments depend both on the presence of the apex and the length of the fragment; smaller fragments have a reduced viability. Several studies reported the high regeneration capacity of the elodeids even from small plant parts [23,24]. The anchoring rate of propagules in sediments was 98% in spring, while it decreased to 73.2% in summer.

In this experiment, the number of branches and roots produced, the dry biomass, and anchoring were lower for propagules cut in summer compared to those generated by one cut in spring. The second hypothesis was not validated. *E. densa* showed comparatively similar apical growth in Spring and Summer. Only the number of buds produced was higher in summer. Therefore, the colonization and regeneration abilities of propagules decreased in summer, despite the maximum growth potential (production of numerous buds that will develop into lateral branches). As the growth phase of *E. densa* ranges from June to September [14,15,18], these results are surprising. One possible explanation for this reduction in *E. densa* establishment abilities in summer was the different environmental conditions between spring and summer. As *E. densa* can use HCO_3-_ for photosynthesis [7], CO_2_ must be far more important for *E. densa* growth rate, and it can thus be concluded that the growth rate was affected by the level of free CO_2_ concentration in water. *E. densa* growth rate could be stimulated in spring while spring water is generally rich in free CO_2_ and therefore directly promotes photosynthesis rate, whereas in summer the level of free CO_2_ was low. Water nutrients have also a potential effect on the regeneration ability of submerged plant fragments [24]. *E. densa* biomass seems to be related to phosphorus concentration in water [25]. A lower phosphorus availability in water in summer could also explain the lowest fitness of summer fragments while *E. densa* was unable to accumulate enough phosphorus in the small fragments to support growth. 

## 5. Conclusions

The cutting date had no effect on *E. densa* biomass. However, two cuts significantly reduced the plant biomass. The removal produces numerous fragments. These fragments have a high survival rate and are capable of generating new stands, especially in the spring. More detailed studies on the dispersal capacities and buoyancy of fragments (maximum capacity and duration of flotation) would help better understand the species’ colonization success and how the use of mechanical control methods can enhance its rate of spread. Because this plant spreads readily through fragmentation and while the fragments have the highest regeneration and colonization abilities in spring, mechanical controls such as cutting should be better used at the end of summer.

## Figures and Tables

**Table 1:** Effect of the season (spring versus summer), timing (beginning or end of the experiment), and their interaction with the traits of *E. densa* fragments (Two-way ANOVA).

	Stem Length			Number Buds			Number Lat Branches			Number Root		
	*df*	*F*	*p*	*df*	*χ*	*p*	*df*	*χ*	*p*	*df*	*χ*	*p*
Season	1	11.31	**<0.0001**	1	329.09	**<0.0001**	1	233.53	**<0.0001**	1	537.79	0.101
timing	1	18.81	**<0.0001**	1	136.13	**<0.0001**	1	121.22	**<0.0001**	1	194.14	**<0.0001**
Season × timing	1	0.01	0.914	1	129.46	**0.010**	1	121.03	0.661	1	166.51	**<0.0001**

## Data Availability

Not applicable.

## Supplementary Materials

The following supporting information can be downloaded at: https://www.mdpi.com/article/10.3390/life13102004/s1: Table S1. Water quality monitoring in the five ponds. The water depth was measured above the pots.
