# Supplementary material for: Impact of Mechanical Removal on the Regeneration and Colonization Abilities of the Alien Aquatic Macrophyte Egeria densa
Source: Life (Basel). 2023 Oct 2;13(10):2004. doi: 10.3390/life13102004 (PMC10608586; doi:10.3390/life13102004)
Supplement: Supplementary file 1 [file life-13-02004-s001.zip › life-2608903-supplementary.pdf]

life

MDPI

*Article*

# Impact of Mechanical Removal on the Regeneration and Colonization Abilities of the Alien Aquatic Macrophyte *Egeria densa*

**Thiébaut G**

**Supplementary table**

**Table S1.** Water quality monitoring in the five ponds. The water depth was measured above the pots.

| | Mesocosm 1 | | | Mesocosm 2 | | | Mesocosm 3 | | | Mesocosm 4 | | | Mesocosm 5 | | |
|---|---|---|---|---|---|---|---|---|---|---|---|---|---|---|---|
| temperature °C | 18.1 | ± | 3.4 | 18.0 | ± | 3.3 | 18.1 | ± | 3.3 | 18.1 | ± | 3.3 | 18.1 | ± | 3.3 |
| conductivity µS/cm | 348.4 | ± | 72.5 | 354.8 | ± | 78.9 | 343.1 | ± | 74.6 | 348.7 | ± | 77.4 | 352.0 | ± | 72.1 |
| pH | 8.5 | ± | 1.0 | 8.4 | ± | 0.9 | 8.5 | ± | 1.0 | 8.4 | ± | 1.2 | 8.3 | ± | 0.9 |
| $O_2$ mg/L | 10.0 | ± | 2.8 | 9.4 | ± | 2.5 | 10.3 | ± | 3.6 | 9.9 | ± | 3.3 | 9.0 | ± | 3.2 |
| Ammonium mgN/L | 0.05 | ± | 0.02 | 0.04 | ± | 0.04 | 0.03 | ± | 0.02 | 0.04 | ± | 0.02 | 0.06 | ± | 0.05 |
| Phosphates mgP/L | 0.6 | ± | 0.7 | 0.3 | ± | 0.1 | 0.3 | ± | 0.5 | 0.5 | ± | 0.7 | 1.1 | ± | 0.8 |
| Nitrates mgN/L | 4.5 | ± | 4.5 | 4.4 | ± | 5.4 | 5.1 | ± | 5.2 | 2.9 | ± | 2.8 | 5.1 | ± | 3.5 |
| depth cm | 29.2 | ± | 1.9 | 31.1 | ± | 1.0 | 31.6 | ± | 1.7 | 32.7 | ± | 0.2 | 31.9 | ± | 0.8 |
